# Endothelin-1 receptor antagonists protect the kidney against the nephrotoxicity induced by cyclosporine-A in normotensive and hypertensive rats

**DOI:** 10.1590/1414-431X20176373

**Published:** 2017-12-11

**Authors:** A. Caires, G.S. Fernandes, A.M. Leme, B. Castino, E.A. Pessoa, S.M. Fernandes, C.D. Fonseca, M.F. Vattimo, N. Schor, F.T. Borges

**Affiliations:** 1 Disciplina de Nefrologia, Departamento de Medicina, Universidade Federal de São Paulo, São Paulo, SP, Brasil; 2 Programa Interdisciplinar em Ciências da Saúde, Instituto de Ciências da Atividade Física e Esporte, Universidade Cruzeiro do Sul, São Paulo, SP, Brasil; 3 Laboratorio Experimental de Modelos Animais (LEMA), Escola de Enfermagem, Universidade de São Paulo, São Paulo, SP, Brasil; 4 Departamento de Enfermagem Clínica e Cirúrgica, Escola Paulista de Enfermagem, Universidade Federal de São Paulo, São Paulo, SP, Brasil

**Keywords:** Genetically hypertensive rats, Acute kidney injury, Cyclosporine-A, Bosentan, Macitentan, Endothelin-1

## Abstract

Cyclosporin-A (CsA) is an immunosuppressant associated with acute kidney injury and chronic kidney disease. Nephrotoxicity associated with CsA involves the increase in afferent and efferent arteriole resistance, decreased renal blood flow (RBF) and glomerular filtration. The aim of this study was to evaluate the effect of Endothelin-1 (ET-1) receptor blockade with bosentan (BOS) and macitentan (MAC) antagonists on altered renal function induced by CsA in normotensive and hypertensive animals. Wistar and genetically hypertensive rats (SHR) were separated into control group, CsA group that received intraperitoneal injections of CsA (40 mg/kg) for 15 days, CsA+BOS and CsA+MAC that received CsA and BOS (5 mg/kg) or MAC (25 mg/kg) by gavage for 15 days. Plasma creatinine and urea, mean arterial pressure (MAP), RBF and renal vascular resistance (RVR), and immunohistochemistry for ET-1 in the kidney cortex were measured. CsA decreased renal function, as shown by increased creatinine and urea. There was a decrease in RBF and an increase in MAP and RVR in normotensive and hypertensive animals. These effects were partially reversed by ET-1 antagonists, especially in SHR where increased ET-1 production was observed in the kidney. Most MAC effects were similar to BOS, but BOS seemed to be better at reversing cyclosporine-induced changes in renal function in hypertensive animals. The results of this work suggested the direct participation of ET-1 in renal hemodynamics changes induced by cyclosporin in normotensive and hypertensive rats. The antagonists of ET-1 MAC and BOS reversed part of these effects.

## Introduction

Cyclosporine-A (CsA) is an agent widely used in transplants, such as kidney transplant (1). It is a cyclic, lipophilic medicine, and has immunosuppressive activity to T cells and other inflammatory cells. It binds to cyclophilin, its cytoplasmic receptor, and the CsA-cyclophilin complex binds to calcineurin, a serine-threonine phosphatase that is calcium- and calmodulin-dependent, inhibiting their ability to dephosphorylate nuclear factors present in the cytosol (2,3) as nuclear factor T cells (NF-ATc). CsA blocks NF-ATc and inhibits the transcription process, preventing the production of these cytokines (4).

The adverse effects, such as nephrotoxicity, hepatotoxicity, hypertension and risk of malignancy, are observed in sub-acute and chronic treatment (5). Renal dysfunction induced by CsA in humans is an independent risk factor for graft loss and mortality after kidney transplantation, and cardiovascular disease is the main cause of death post-transplantation. CsA use has been described to be associated with chronic kidney disease and end stage renal disease. Diabetes mellitus and hypertension are the main risk factors to renal disease leading to kidney transplantation. It is reasonable to suggest that both events can superimpose. In rats, the tubular atrophy and irreversible interstitial fibrosis, initially compromising the medullary rays and after the renal cortex, are observed (6).

The renal vascular injury induced by CsA involves afferent arterioles and small arteries with resultant increase in renal vascular resistance, decrease in renal blood flow and glomerular filtration (7). The arteriolar vasoconstriction induced by cyclosporine-A is due to the activation of the renin-angiotensin system (7–9). Attempts to prevent acute toxicity with angiotensin converting enzyme blockers or angiotensin receptors antagonists showed conflicting results. Burdmann et al. (6), demonstrated that it is possible to prevent interstitial fibrosis induced by CsA with the use of losartan and enalapril. The decrease in renal blood flow caused by chronic vasoconstriction may be the stimulus for the fibrotic process in the renal interstitial as well as for glomerular sclerosis. However, chronic renal ischemia can also lead to interstitial fibrosis. Thus, the use of vasodilation agents can prevent or reverse renal lesion induced by CsA.

Endothelin peptides are produced in various tissues, and they act as modulators of vascular tone, cell proliferation and hormone production (10). Endothelin-1 (ET-1) is mainly related to endothelial dysfunction, but other cells are also capable of producing the ET-1, such as vascular smooth muscle cells and mesangial cells of the kidney (11). Stimuli as ischemia, hypoxia or shear stress of blood vessels induce the production and secretion of ET-1 (10,11).

ET-1 exerts its function in the target cell via two receptors, ETA and ETB (11). ETA receptor activation is primarily responsible for vasoconstriction, cell proliferation, proteinuria and the induction of renal fibrosis induced by endothelin-1. Plasma and renal ET-1 levels are elevated in diabetic patients and mediate diabetic nephropathy. Differently, ETB receptor activation releases vasodilating substances such as prostacyclin and nitric oxide, leading to vasodilation first and then vasoconstriction. These are present in greater quantities in endothelial cells (10,11).

ET-1 can also be involved in the nephrotoxicity induced by CsA. *In vivo* administration of high doses of CsA in mice was associated with a significant increase in the ET-1 production, increase in renal vascular resistance, reduction of glomerular filtration and the development of hypertension. In humans, the association between high systemic levels of ET-1 with the administration of high doses of CsA was observed, suggesting that this relationship may also be observed clinically (10).

Several ET-1 antagonists were developed. Their principal therapeutic use is the treatment of idiopathic pulmonary fibrosis and pulmonary arterial hypertension, reducing morbidity and mortality in patients with pulmonary hypertension (12,13). However, the effect of ET-1 antagonists in renal blood flow or renal fibrosis induced by the use of CsA in hypertensive animals was poorly investigated.

The present study compared the effect of ET-1 antagonist bosentan and the new antagonist macitentan, also called actelion-1 or ACT-064,922, in the nephrotoxicity induced by CsA in hypertensive rats.

## Material and Methods

### Animal treatment

The experimental protocol was approved by the Ethics Committee of the Universidade Cruzeiro do Sul, also in agreement with the Brazilian guidelines for scientific animal care and use (14,15).

Male Wistar rats and genetically hypertensive rats (SHR), weighing 200–250 g, were divided into groups of 10 rats. The animals in the control group were maintained under controlled conditions during the experimental protocol. The CsA group received daily intraperitoneal injections of CsA (Sigma, USA), 40 mg/kg, during the last 15 days. The rats in the CsA and bosentan group (CsA+BOS) were treated with intraperitoneal injections of CsA (40 mg/kg) as well as daily intraperitoneal injections of bosentan (5 mg/kg). The CsA and macitentan group (CsA+MAC) was treated with intraperitoneal injections of CsA (40 mg/kg) as well as daily macitentan (25 mg/kg) administration by gavage.

The animals were allowed free access to tap water and low sodium food initiated 3 days before and during the experimental protocol. The low sodium diet composed of carbohydrate, vitamins and amino acids supplement (Aminomix, Vetnil, Brazil) was used to potentiate the nephrotoxic effect of CsA, according to Burdmann et al. (6). At 0 (basal) and 15 days (post-treatment) after the beginning of experimental protocols, blood samples were collected from the lateral tail vein, and the animals were maintained in metabolic cages over 24 h for urine collection. In some experiments, the urine volume after at least 3 days of hyposodic diet (post-diet) was collected and quantified. The animals were killed 15 days after the beginning of the experimental protocol, and both the right and left kidneys were removed for immunohistochemistry analysis. Biochemical parameters in the plasma and urine samples were determined.

### Biochemical analysis

The levels of blood creatinine and blood urea were assayed spectrophotometrically according to standard procedures by using commercially available diagnostic kits (Labtest Diagnostica, Brazil). Creatinine was determined by a colorimetric method based on the Jaffé reaction (16) and the creatinine clearance was assessed. Urea was determined using a colorimetric assay based on urease activity (17). Levels of creatinine and urea are reported in mg/dL. Urine sodium concentrations in urine were determined with a Micronal B462 flame photometer (Brazil). Sodium excretion are reported as percentage of the basal sodium excretion.

### Kidney hemodynamics

On the last day of the experimental protocol, 10 animals per group were anesthetized with ketamine (100 mg/kg) and xylazine (10 mg/kg) intraperitoneal injections (Agribands, Brazil) for measurement of renal blood flow (RBF). The left renal artery was isolated and surrounded by an ultrasonic flowmeter probe (T402, Transonic Systems Inc., USA) to measure blood vessel flow. Mean arterial blood pressure (MAP) was recorded via catheterization of the femoral artery and renal vascular resistance (RVR) was calculated by the following formula: RVR (mmHg·mL^−1^·min^−1^) = MAP (mmHg)/RBF (mL/min). At the end of the experimental protocol, blood collection was performed by puncturing the abdominal aorta.

### Oxidative stress studies

Urinary peroxides were determined by the ferrous oxidation of xylenol orange version 2 (FOX-2) method. Ferrous iron is oxidized to ferric iron by peroxides contained in the samples. Xylenol orange reagent shows a high selectivity for the Fe^3+^ ion, producing a bluish-purple complex whose absorbance can be measured at 560 nm (A = 4.3×10^4^ M/cm). Aliquots (200 µl) of plasma were mixed with 1.8 mL of xylenol orange reagent. The data were corrected for gram urinary creatinine and are reported as nmol/g (18). The lipid peroxidation levels of malondialdehyde were determined by methods measuring thiobarbituric acid-reactive substances (TBARS). For the quantification, 0.4 mL of urine sample with 0.6 mL water was added to a reaction mixture consisting of 1.0 mL 17.5% trichloroacetic acid (TCA) and 1.0 mL 0.6% thiobarbituric acid. This mixture was heated in a water bath at 95°C for 20 min, the solution was removed from the water bath and cooled on ice, followed by the addition of 1.0 mL 70% TCA. The solution was homogenized and incubated for 20 min. Finally, the solution was read in a spectrophotometer at 534 nm (A = 1.56×10^5^ M/cm). The data are reported as nmol/g creatinine (19).

### Immunohistochemistry

The kidneys were dissected along the non-hilar axis and fixed in 10% phosphate buffered formalin (Erviegas, Brazil). Kidney sections were fixed with 4% buffered paraformaldehyde and then embedded in paraffin (Erviegas). Next, 4-μm thick sections were prepared. The kidney slices were deparaffinized and rehydrated. Endogenous peroxidase activity was blocked with 5% H_2_O_2_ in absolute methanol for 10 min at room temperature. To expose the antigens, kidney sections were boiled in a target retrieval solution [1 mmol/L tris(hydroxymethyl)aminomethane (Tris), pH 9.0, with 0.5 mM ethylene glycol tetraacetic acid (EGTA)] for 10 min. Nonspecific binding was prevented by incubating the sections in phosphate buffered saline (PBS) containing 1% bovine serum albumin (BSA), 0.05% saponin, and 0.2% gelatin. Sections were incubated with primary antibodies against ET-1 (1:200, rabbit anti-rat; ABCAM, MA, USA) and angiotensin-2 (1: 200, rabbit anti-rat IgG; Santa Cruz Biotechnology, USA). Protein expression was measured using a streptavidin peroxidase kit (Dako, USA). Sections were stained with diaminobenzidine for antibody detection and then counterstained with hematoxylin. Signals in negative control sections were absent. Digital photomicrographs were taken through a Leica DM 1000 upright microscope connected to a workstation computer through the Leica DFC 310 FX, LAS 3.8 Microscope Camera (Leica, Switzerland). Ten photomicrographs along the kidney cortex were taken and the light brown staining was quantified (LAS software, version 3.8) and averaged for each rat. The data are reported as percentage of stained area.

### Statistical analysis

Results are reported as a means±SE. Data were analyzed by one-way analysis of variance (ANOVA) followed by the Tukey test or Student's *t*-test. P<0.05 was considered statistically significant.

## Results

### Physiological parameters

**Figure 1.**
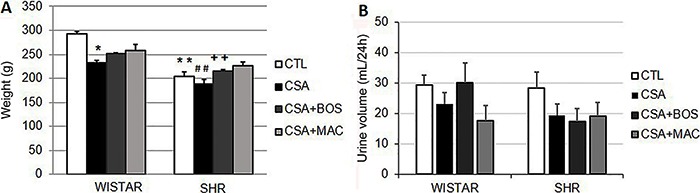
Quantitative analyses of *A*, body weight, and *B*, urine volume in Wistar rats (normotensive) and genetically hypertensive rats (SHR) in the control group (CTL), in rats receiving cyclosporine-A (CsA) or receiving CsA and treated with bosentan (CsA+BOS) or macitentan (CsA+MAC) for 15 days. Data are reported as means±SE. The significance level for a null hypothesis was set at 5% (P<0.05). ^*^P<0.05, compared to the CTL group; ^**^P<0.05 compared to the respective normotensive group; ^##^P<0.05 compared to the respective CsA normotensive group. ^++^P<0.05 compared to the respective CsA+BOS normotensive group (ANOVA followed by the Tukey test or Student's *t*-test).

At the end of the experimental protocol, the hypertensive animals of the experimental groups CTL, CsA, CsA+BOS had significantly lower weight than the animals of the respective normotensive groups; the CSA+MAC group had no difference in weight compared to their normotensive group (Figure 1A).

In normotensive animals, treatment with cyclosporin significantly decreased the weight of the animals compared to the control group. This decrease was not observed in animals treated concomitantly with CsA and BOS or MAC.

In hypertensive animals, although treatment with CsA showed a tendency to decrease weight, there was no significant difference in the weight of the CsA, CsA+BOS and CsA+MAC groups compared to the control group (Figure 1A).

There was an increase in the urinary volume after the onset of the hyposodic diet in normotensive (basal: 13.1±1.5 and post-diet: 48.0±4.6 mL) and hypertensive (basal: 12.9±1.8 and post-diet: 36.4±3.2 mL) rats. This was expected since sodium is the main ion responsible for the hydroelectrolytic balance in the organism.

In the post-treatment period, there was no significant difference in urinary volume between experimental groups in normotensive or hypertensive animals (Figure 1B).

### Renal hemodynamics

**Figure 2.**
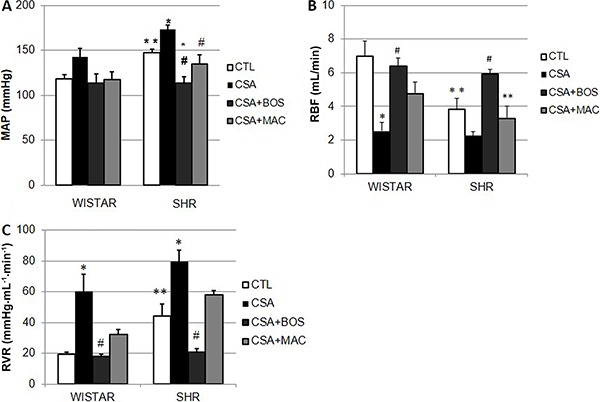
Quantitative analyses of *A,* medium arterial blood pressure (MAP); *B*, renal blood flow (RBF), and *C*, renal vascular resistance (RVR) in Wistar (normotensive) and genetically hypertensive (SHR) animals in the control group (CTL), rats receiving cyclosporine-A (CsA) or receiving CsA and treated with bosentan (CsA+BOS) or macitentan (CsA+MAC) for 15 days. Data are reported as means±SE. The significance level for a null hypothesis was set at 5% (P<0.05). ^*^P<0.05, compared to the CTL group; ^**^P<0.05, compared to the respective normotensive control group; ^#^P<0.05, compared to the CsA group (ANOVA followed by the Tukey test or Student's *t*-test).

MAP was analyzed at the end of the experimental protocol by means of a probe in the femoral artery. The results are presented in Figure 2.

The MAP of the hypertensive animals was higher than the normotensive animals, as expected. Treatment with CsA further increased the MAP in the normotensive (CTL: 118±5; CsA: 143±9 mmHg) and hypertensive animals (CTL: 147±4; CsA: 173±5 mmHg) in relation to the control. Concomitant treatment with cyclosporin and BOS or MAC decreased MAP in the normotensive (CsA+BOS: 113±10, CsA+MAC: 117±8 mmHg) and hypertensive (CsA+BOS: 113±7; CsA+MAC: 135±10 mmHg) animals compared to the cyclosporine-treated group. However, this effect was only significant in hypertensive animals (Figure 2A).

Figure 2B shows RBF analyzed in normotensive and hypertensive animals at the end of the experimental protocol. There was a significant decrease in RBF in hypertensive animals compared to normotensive animals in the control group.

Both the hypertensive (CTL: 6.9±0.9 *vs* CsA: 2.5±0.4 mL/min) and normotensive (CTL: 3.8±0.6 *vs* CsA: 2.2±0.2 mL/min) animals treated with CsA showed a decrease in renal blood flow compared to the respective control animals. However, only in normotensive (P<0.05) animals, not in hypertensive animals (P=0.05), this decrease was significant (Figure 2B). Treatment with BOS reversed the decrease in CsA-induced RBF in normotensive (CsA+BOS: 6.38±0.6 mL/min) and hypertensive (CsA+BOS: 5.95±0.8 mL/min) animals. MAC treatment showed the same pattern, increasing RBF in normotensive (CsA+MAC: 4.7±1.1 mL/min, P=0.05) and hypertensive (CsA+MAC: 3.3±0.8 mL/min, P>0.05) animals but this result was not significant.

Figure 2C shows the RVR measured in the renal artery of hypertensive and normotensive animals. We observed that RVR was significantly higher in the hypertensive control group. Treatment with CsA significantly increased RVR in both normotensive (CTL: 19.3±1.6, CsA: 60.5±10.9 mmHg·mL^-1^·min^-1^) and hypertensive animals (CTL: 44.2±10.0; CsA: 79.8±8.4 mmHg·mL^-1^·min^-1^) and concomitant treatment with BOS, but not with MAC, reversed the increase in RVR induced by CsA.

### Renal function

**Figure 3.**
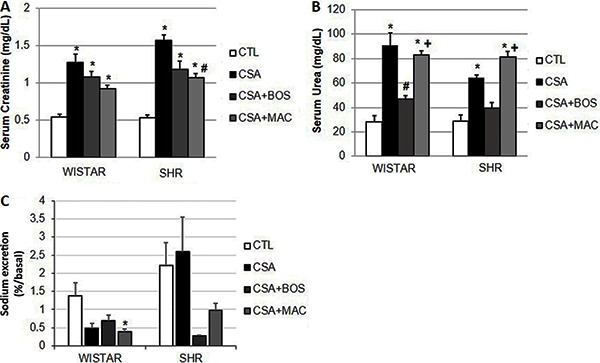
Quantitative analyses of *A*, plasmatic creatinine, *B*, plasmatic urea, and *C*, sodium excretions in Wistar (normotensive) and genetically hypertensive (SHR) rats in the control group (CTL), receiving cyclosporine-A (CsA) or receiving CsA and treated with bosentan (CsA+BOS) or macitentan (CsA+MAC) for 15 days. Data are reported as means±SE. The significance level for a null hypothesis was set at 5% (P<0.05). ^*^P<0.05, compared to the CTL group; ^#^P<0.05, compared to the CsA group. ^+^P<0.05, compared to the CsA+BOS group (ANOVA followed by the Tukey test or Student's *t*-test).

Renal function was assessed by plasma urea and creatinine (Figure 3). There was a significant increase in plasma creatinine in animals treated with CsA in the post-treatment period in normotensive (CTL: 0.54±0.03; CsA: 1.27±0.11 mg/dL) and hypertensive (CTL: 0.79±0.15, CsA: 1.57±0.07 mg/dL) animals compared to the control group. Concomitant treatment with BOS or MAC in normotensive (BOS: 1.07±0.07, MAC: 0.92±0.4 mg/dL) or hypertensive (BOS: 1.17±0.10, MAC: 1.07±0.05 mg/dL) animals did not alter this pattern. One exception was CsA+MAC in hypertensive animals where creatinine was significantly decreased compared to the CsA group. (Figure 3A).

Figure 3B shows the plasma urea concentration in the CsA treated animals and concomitantly treated with BOS and MAC. Corroborating creatinine results, there was a significant increase in plasma urea in the CsA treated animals in comparison to the respective normotensive (CTL: 28.1±5.1, CsA: 91.0±10.2 mg/dL) and hypertensive (CTL: 28.7±5.1, CsA: 64.4±2.3 mg/dL) control groups. Concomitant treatment with BOS but not with MAC significantly decreased plasma urea in normotensive animals (BOS: 46.8±10.2; MAC: 83.1±3.1 mg/dL) and hypertensive animals (BOS: 39.6±2.2; MAC: 81.5±4.5 mg/dL).

Figure 3C shows the sodium concentration in the urine of normotensive and hypertensive animals. In normotensive animals, the CsA-treated animals and BOS or MAC concomitantly treated with CsA animals showed a decreased urinary sodium excretion compared to the control group, but this effect was only significant in the CsA+MAC group.

In hypertensive animals, treatment with CsA concomitantly with BOS and MAC decreased urine sodium, but this effect was not significant (Figure 3C).

### Oxidative stress studies

Oxidative stress was analyzed in urine by lipid peroxidation by urinary peroxides and tiobarbituric reactive metabolites. The CsA group presented higher rates of oxidative metabolites, urinary peroxides and TBARS compared to the CTL group in normotensive and hypertensive rats. Endothelin antagonists BOS and MAC blunted this effect (Table 1). Although there was a trend, no significant difference in TBARS was observed in CsA group compared to the CTL group in hypertensive animals.

**Table 1.** Reactive oxygen species metabolites in Wistar and genetically hypertensive rats (SHR).

Groups	Urinary peroxides (nmol/g urinary creatinine)		TBARS (nmol/g urinary creatinine)	
	Wistar	SHR	Wistar	SHR
CTL	4.7±3.4	5.3±3.5	0.35±0.14	0.45±0.30
CsA	45.6±0.3*	16.0±3.1	1.48±0.74*	1.10±0.44
CsA+BOS	4.8±2.5#	5.3±2.9#	0.42±0.09#	0.65±0.42
CsA+MAC	3.4±1.3#	2.7±1.5#	0.69±0.20#	0.68±0.08

Data are reported as means±SE. TBARS: thiobarbituric acid-reactive substances; CsA: cyclosporin-A; BOS: bosentan; MAC: macitentan.

* P<0.05 *vs* CTL.

# P<0.05 vs CsA (ANOVA followed by the Tukey test or Student's t-test).

### Immunoblotting

**Figure 4.**
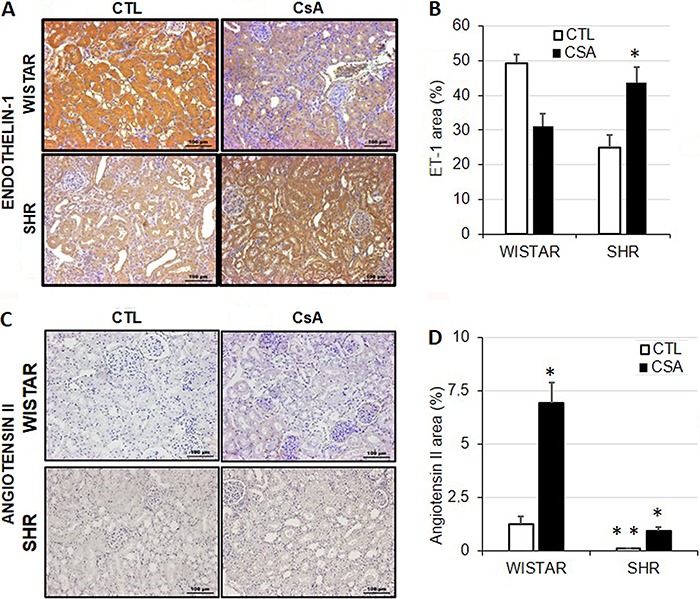
*A*, Light microscopy of kidney sections immunostained for endothelin-1 (ET-1, 200×) and (C) angiotensin II (200×) in control rats (CTL) and rats receiving cyclosporine-A (CsA). *B*, Quantitative analyses of kidney sections stained for ET-1 and (*D*) angiotensin II. Data are reported as means±SE. The significance level for a null hypothesis was set at 5% (P<0.05). ^*^P<0.05, compared to the CTL group, and ^**^P<0.05, compared to the respective normotensive group (ANOVA followed by the Tukey test or Student's *t*-test). SHR: genetically hypertensive rats.

Figure 4A and B show ET-1 and angiotensin II immunostaining in the renal cortex of normotensive and hypertensive animals treated with CsA. ET-1 was significantly lower in the renal cortex of control hypertensive animals (CTL: 24.9±3.7%) compared to control normotensive animals (CTL: 49.4±2.3%; Figure 4B). After treatment with CsA, this pattern changed, and a significant increase in the labeling for ET-1 occurred in hypertensive (CsA: 43.8±4.3%) but not in normotensive animals (CsA: 31.1±3.7%). Taken together, this result showed that the production of ET-1 in the renal cortex increased in hypertensive animals after treatment with CsA.

Figure 4C and D show the immunoblotting for angiotensin II in the renal cortex of normotensive and control hypertensive animals and animals treated with CsA. Angiotensin II was significantly lower in the renal cortex of hypertensive animals (CTL: 0.11±0.03%) compared to normotensive animals (CTL: 1.25±0.36%). After treatment with CsA, there was a significant increase in the immunostaining in normotensive (CsA: 6.9±0.9%) and hypertensive animals (CsA: 0.9±0.2%).

## Discussion

The side effects associated with the prolonged treatment with CsA (nephrotoxicity, hypertension) limit their use. Alternative treatments such as the use of macitentan to CsA-induced nephrotoxicity can attenuate the problem. CsA exhibits a distinct pattern of toxicity characterized by acute, subacute and chronic toxicity (20).

In this study, renal lesions were observed in rats in subacute toxicity. This was associated with an increased serum creatinine and a decrease in glomerular filtration rate, arteriolar lesions and arterial hypertension. In our experimental conditions, we observed an increase in MAP and RVR with a decrease in RBF in hypertensive animals when compared to normotensive animals. Treatment with CsA for 15 days caused an increase in MAP and RVR with a decrease in RBF in both hypertensive and normotensive animals. The effect of CsA and other immunosuppressive agents on systemic and renal hemodynamics has been demonstrated. The vasoconstrictive effect of CsA has already been well characterized in other studies in the literature (6,8,21,22). Interestingly, concomitant treatment with BOS prevented CsA-induced hemodynamic changes in normotensive animals, but mainly in hypertensive animals, since BOS not only reversed the effects of CsA on hypertensive animals, but also brought the values of MAP, RVR and RBF to values close to normotensive animals. This result suggests that the effect of ET-1 induced by CsA was more pronounced in hypertensive animals. Interestingly, immunohistochemistry for ET-1 corroborates this effect. In addition, the involvement of ET-1 in immunosuppressive-induced nephrotoxicity has been demonstrated previously with the use of tracrolimus (FK-506) (23). In this nephrotoxicity model, renal vasoconstriction induced by FK-506 was also observed.

We observed an increase in ET-1 production in the renal cortex mainly in hypertensive animals. In normotensive animals, CsA did not induce an increase in ET-1, but induced a significant increase in angiotensin II, which has a renal vasoconstricting action and increases MAP. The involvement of angiotensin II in renal vasoconstriction and in the decrease of renal function induced by CsA has also been shown in the literature (7,24).

MAC at the doses used in the present study decreased MAP and increased RBF in hypertensive animals, where endothelin-1 production was prominent. A similar pattern was observed for BOS. Protective effects of novel ET-1 receptor antagonists have been reported in diabetic nephropathy and renovascular disease in doses similar to those used in our experimental model (25,26).

In renal function, CsA treatment increased plasma creatinine and urea in normotensive and hypertensive animals. Concomitant treatment with BOS and MAC did not alter creatinine, but BOS significantly decreased urea. In our experimental conditions, creatinine may be a marker of renal function less sensitive to the effect of CsA. Perhaps another renal function marker should be analyzed, such as insulin clearance, but this still needs to be determined.

We did not observe alterations in the production of urine in the animals submitted to our experimental conditions. No difference was observed in the weight of the animals among the experimental groups at the end of the protocol, although the hypertensive animals had significantly lower weight when compared to normotensive animals. Excretion of sodium in the urine decreased in the animals after treatment with CsA in the absence or presence of BOS or MAC, but this effect was only significant in the group of normotensive animals treated with CsA and MAC. It is interesting to note that these results may have undergone variations because of the hyposodic diet.

The administration of CsA in Wistar and SHR rats induced reactive oxygen species generation by enhancement of urinary peroxides and TBARS. BOS and MAC reduced this effect probably by increasing the antioxidant capacity in the kidney. Several studies have described the crucial role of reactive oxygen species in drug-induced nephropathy, including CsA (27,28).

The results of this study suggested the direct participation of ET-1 in cyclosporine-induced changes in renal hemodynamics in rats, especially in hypertensive animals. We used two drugs considered antagonists of endothelin-1, bosentan-Ro470203, a non-selective antagonist of ET-A and ET-B receptors, and the non-specific endothelin receptor antagonist macitentan. The effects of ET-1 antagonists were not significantly different between normotensive and hypertensive animals. However, at doses used in the present study, bosentan appeared to be better at reversing hemodynamic changes and renal function induced by CsA, mainly in hypertensive animals, but macitentan could be an alternative to prevent cyclosporine-A toxicity.
